# Supplementary material for: Colibactin-associated mutations in the human colon appear to reflect anatomy and early exposure, not oncogenesis
Source: medRxiv. 2026 Apr 15:2026.04.13.26350783. Preprint. [Version 1] doi: 10.64898/2026.04.13.26350783 (PMC13127518; doi:10.64898/2026.04.13.26350783)
Supplement: Supplement 2 [file media-2.pdf]

**Overlapping signatures**	**Biological significance**	**Tumor trend**	**Match in regional trends?**	**Interpretation**
SBS1	Spontaneous deamination; clock-like	Decreased right-to-left	No; more comparable in at-risk clinical group	Hypermutability associated with microsatellite instability absent in our data; age or advanced clinical state may influence mutagenesis
SBS5	Clock-like; associated with cell division/aging	Decreased right-to-left	No	Hypermutability…
SBS18	Oxidative damage	Decreased right-to-left	**Yes**	Oxidative damage is elevated in the proximal colon
ID1	Slippage during replication; clock-like	Decreased right-to-left	No; more comparable in at-risk clinical group	Hypermutability… age or clinical state…
ID2	Slippage during replication; clock-like	Decreased right-to-left	Slightly**;** more comparable in at-risk clinical group *and* in subclonal mutations	Hypermutability… age or clinical state…
ID18	Colibactin	Increased right-to-left	**Yes**	Colibactin mutagenesis enriched in the distal/rectal colon

Supplementary Table 1: Contextualization of Cornish et al. signatures overlapping with this study.

References associating hypermutability with microsatellite instability in the proximal colon:

1. (Farmanbar et al. 2023).
2. (Kim et al. 2024).
3. (Xu et al. 2024).
4. (Ward et al. 2025)

Anatomic term	Statistical Test	Mutation subtype	Adj. p-value (*p-val.*)
Side (Right vs left)	Paired Wilcoxon test	C>A	0.380 (*0.380*)
		C>G	0.051 (*0.014*)
		***C>T**	**0.027 (*0.003*)**
		CpG>TpG	0.083 (0.059)
		T>A	0.051 (*0.029*)
		T>C	0.051 (*0.029*)
		T>G	0.280 (*0.240*)
Region (Cecum, ascending colon, transverse colon, descending colon, sigmoid colon, rectum)	Kruskal-Wallis test	****C>A**	**0.008 (*0.003*)**
		***C>G**	**0.042 (*0.030*)**
		C>T	0.081 (*0.070*)
		CpG>TpG	0.215 (*0.215*)
		*****T>A**	**< 0.001 (< *0.001*)**
		****T>C**	**0.001 (< *0.001*)**
		***T>G**	**0.031 (*0.018*)**

Supplementary Table 2: Non-GLM statistical overview of mutation subtypes by location. All p-values adjusted by Benjamini-Hochberg correction. Significant adjusted p-values are bolded alongside mutation subtype, with asterisks indicating significance level: *** < 0.001, ** < 0.01, * < 0.05.

Source	Study description	Included	Excluded	MAF?
Our study	Whole-genome sequencing of 125 **“normal” crypts** from 21 individuals	**116** crypts (**21** patients)	Nine crypts<br>- 6 with coverage < 10<br>- 3 crypts with total SNV burden < 500	Yes
Lee-Six et al., 2019	Whole-genome sequencing of 445 **“normal” crypts** from 42 individuals<br>-	*Quality comparisons:* **445** crypts (**42** patients)<br><br>*Genotyped (340 total):* **301** crypts (**38** patients)<br>- 113 crypts from 15 patients with CRC<br>- 188 crypts from 23 healthy individuals	*Not genotyped:* 2 patients (105 crypts); too many samples to joint call with freebayes<br><br>*Genotyped (340 total):*<br>- 16 crypts removed for being ileum (small intestine)<br>- 12 crypts removed for having coverage < 10<br>- 11 crypts removed for having <500 crypt-specific SNVs	In genotyped data
Cornish et al., 2024	Whole-genome sequencing of 2,023 colorectal cancer samples (**tumors**) from participants in the UK 100,000 Genomes Project	**766** regionally annotated tumors<br>- Cecum (n=158)<br>- Ascending colon (n=79)<br>- Transverse colon (n=60)<br>- Descending colon (n=34)<br>- Sigmoid colon (n=262)<br>- Rectum (n=173)	140 tumors at locations not in our dataset<br>- Hepatic flexure (n=21)<br>- Splenic flexure (n=21)<br>- Rectosigmoid (n=98)<br><br>1,117 tumors without anatomic site annotated	No, public data only
Diaz-Gay et al., 2025	Whole-genome sequencing of 981 colorectal cancers (**tumors**) from 11 countries on 4 continents	**802** microsatellite-stable tumors<br>- Proximal (n=189)<br>- Distal (n=298)<br>- Rectal (n=315)	153 microsatellite-unstable tumors; 26 tumors otherwise classified	No, public data only

Supplementary Table 3: Data manifest for study sources used in manuscript.
